# Supplementary material for: ALPHA/BETA HYDROLASE DOMAIN CONTAINING 5 SUPPORTS GLUCOSE-STIMULATED-LIPOLYSIS AND INSULIN SECRETION IN PANCREATIC BETA CELLS
Source: bioRxiv. 2025 May 23:2025.05.21.655350. Preprint. [Version 1] doi: 10.1101/2025.05.21.655350 (PMC12258721; doi:10.1101/2025.05.21.655350)
Supplement: Supplement 1 [file media-1.pdf]

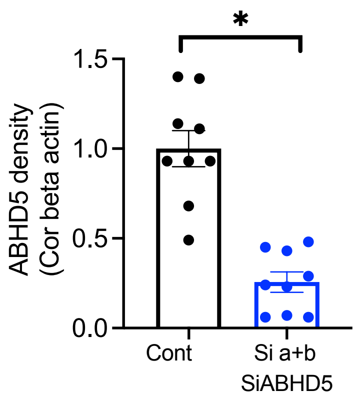


Supplementary Figure 1: INS-1 cells were transfected with non-targeting SiRNA (Cont) or the combination of two SiRNA against ABHD5 (Si a+b). Western blot of ABHD5 and beta-actin was performed and band intensity of ABHD5 corrected for beta-actin was expressed taking average value for Cont in each experiment as 1. n= 9. Mean ± sem. *; $p<0.05$ by Student's t test.
